# Data-driven closed-loop health education: constructing an integrated assessment–intervention–feedback pathway for digital COPD management—a perspective

**DOI:** 10.3389/fpubh.2026.1806393

**Published:** 2026-04-13

**Authors:** Yu Zhou, Ting Wang, Yi-Di Wang, Guo-Ru Li, Pei Sun, Yong-Li Li, Le-Le Yao, Shu-Ling Liang

**Affiliations:** 1 Department of Nursing Care, Baoji People’s Hospital, Baoji, China; 2 Department of Respiratory and Critical Care Medicine, Baoji People’s Hospital, Baoji, China

**Keywords:** chronic obstructive pulmonary disease, closed-loop system, data-driven care, digital health management, health education, patient self-management, personalized intervention

## Abstract

Chronic obstructive pulmonary disease (COPD) imposes a substantial global health burden, yet conventional management often fails to sustain patient engagement or deliver personalized education. Digital technologies now enable continuous, multidimensional data collection, creating an opportunity to transition from standardized care to dynamic, tailored health support. This perspective article proposes a data-driven “assessment–intervention–feedback” closed-loop framework for COPD health education. By integrating multisource data and leveraging intelligent analytics, the system personalizes educational content, optimizes delivery timing, and iteratively refines strategies based on real-world feedback. The framework enhances the relevance and long-term effectiveness of patient education, fostering improved self-management and health outcomes. We further outline its core components, implementation pathways, and key challenges—such as digital equity and clinical integration—to guide the development of scalable, evidence-based digital education solutions in COPD care.

## 1 Introduction

### 1.1 Disease burden and management challenges of chronic obstructive pulmonary disease (COPD)

COPD is a prevalent chronic respiratory disorder characterized by persistent airflow limitation, contributing to a substantial global health and economic burden due to its high morbidity and mortality (1, 2). Traditional COPD management, which relies predominantly on periodic clinical visits, faces considerable limitations (3). These include difficulties in maintaining long-term patient adherence, gaps in the ongoing monitoring of symptoms and exacerbation risks, and a reliance on standardized protocols that often fail to address individual patients’ evolving needs (4). Within this model, patient education—a fundamental component of care—is frequently delivered in an episodic and fragmented manner (5). This approach lacks continuity, systematic reinforcement, and integration with real-world patient data, ultimately limiting its effectiveness in supporting sustained self-management.

### 1.2 Emergence and potential of digital health management

Advances in digital technologies—such as mobile health platforms, wearable devices, and data analytics—present new opportunities for transforming chronic disease care. These tools enable the continuous, passive collection of multidimensional data on patient physiology, symptoms, medication use, and behavior (6). This data-driven foundation facilitates a shift from generalized, static management strategies toward personalized, dynamic health interventions (7). Specifically for health education, digital systems allow for the tailored generation, timely delivery, and iterative adaptation of educational content (8). This ensures that guidance is relevant to the individual’s immediate health status, personal risks, and daily context, moving beyond one-size-fits-all information toward precision patient support (9).

### 1.3 Study perspective and objectives

**Figure 1:**
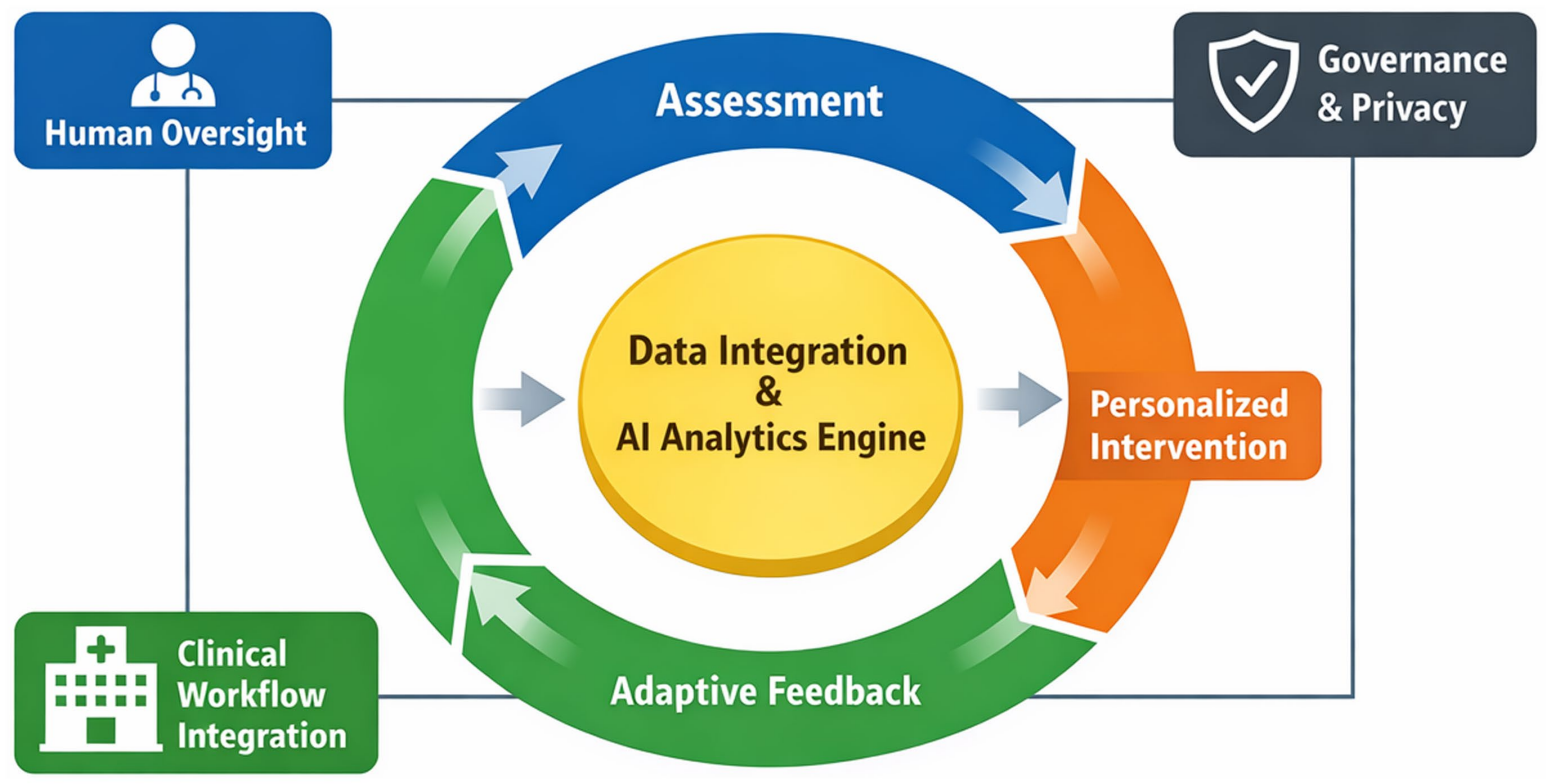
Conceptual architecture of the data-driven assessment-intervention-feedback closed-loop system for digital COPD management.

To address the current fragmentation in health education and leverage the potential of digital tools, this perspective article proposes an “assessment-intervention-feedback” closed-loop framework as the core structure for digital health education in COPD. This framework is designed to establish an iterative, adaptive system: it begins with a continuous assessment of the patient’s knowledge, behaviors, and clinical status using collected data; proceeds to personalized digital interventions based on this assessment; and then employs feedback from patient engagement and health outcomes to evaluate and refine subsequent actions. To enhance the conceptual clarity of this iterative cycle, Figure 1 adopts a simplified circular structure. Its directional flow has been deliberately streamlined to avoid overlapping pathways, thereby intuitively conveying the continuous yet orderly process of data integration and adaptive optimization that underpins the framework. Building upon this conceptual model, this perspective article aims to examine the conceptual underpinnings and essential components—including technological infrastructure, educational content design, and engagement mechanisms—of this closed-loop pathway (Figure 1). Furthermore, it will discuss key implementation challenges, such as data interoperability, integration into clinical workflows, and ensuring equitable access, thereby offering a structured approach for developing more effective and scalable digital education solutions for COPD management.

## 2 Theoretical framework: core logic of “assessment–intervention–feedback” closed loop

### 2.1 Theoretical foundations of closed-loop management

The “assessment-intervention-feedback” framework is grounded in the “Plan-Do-Check-Act” cycle from quality management, which emphasizes continuous improvement through iterative monitoring and adjustment (10–12). This concept has been adapted in health care to form a patient-centered, data-driven approach. Its core principles are twofold: first, it places the patient at the center, ensuring all activities address individual needs and experiences (10, 11); second, it commits to ongoing refinement, using structured cycles to dynamically optimize management strategies rather than applying static, one-time solutions (13). This methodology provides a robust foundation for the long-term, adaptive management required for COPD.

### 2.2 Components of the digital health education closed loop for COPD

The system operates through three interconnected, cyclical phases. The Assessment Phase involves the systematic collection of multidimensional data—including physiological metrics, behavioral patterns, cognitive states, and environmental factors—using digital tools such as wearable devices and mobile applications (14). Based on this assessment, the Intervention Phase delivers tailored health education, behavioral guidance, and facilitates structured communication between patients and providers (15). Finally, the Feedback Phase continuously monitors outcomes, analyzes new data to evaluate intervention effectiveness, and informs adjustments to subsequent assessment and intervention strategies, thereby completing the iterative cycle (16). The key components, data sources, digital strategies, and expected outcomes of each phase are summarized in Table 1.

**Table 1:** Key components, data sources, digital strategies, and expected outcomes across the closed-loop framework.

Closed-loop phase	Key data sources	Digital strategies	Educational focus	Expected outcomes
Assessment	Wearables, PROMs, environmental sensors	Data integration, AI risk stratification	Knowledge gaps, self-management capacity	Personalized patient profile
Intervention	Assessment outputs	Tailored content delivery, tele-support	Medication adherence, exercise, symptom recognition	Improved engagement and behaviors
Feedback	Engagement metrics, clinical outcomes	Real-time monitoring, trend analysis	Reinforcement and adjustment	Continuous optimization

PROMs, patient-reported outcome measures; AI, artificial intelligence.

### 2.3 Logic of data-driven integration

As depicted in Figure 1, this data-driven integration functions as the operational core of the closed loop, linking each phase through a structured and visually coherent cycle rather than a multi-directional convergence of processes. Data flow functions as the central mechanism of the closed loop, connecting all phases in a continuous cycle of input, analysis, action, and reevaluation (17). During the assessment phase, raw data is integrated and analyzed to generate actionable insights (17). These insights drive personalized interventions, which in turn produce new outcome data. This new data is captured as feedback, analyzed, and used to refine the next iteration of the cycle (17). Key technologies enable this process: the Internet of Things supports continuous data capture, artificial intelligence algorithms identify patterns and personalize recommendations, and visualization tools present information clearly to support decision-making (18–20). Collectively, these components transform the closed loop from a manual process into an intelligent, adaptive management system that enhances both the precision and efficiency of COPD care.

## 3 Implementation pathway: digital strategies for Core components

### 3.1 Establishing a multidimensional assessment system

An effective digital health education system begins with a comprehensive and dynamic assessment of the patient. This is achieved by integrating diverse data sources. Objective metrics—such as lung function from portable devices, symptom logs via mobile apps, physical activity from wearables, and environmental data from air quality sensors—provide continuous physiological and contextual insights (21). Complementing these, subjective data are gathered through electronic patient-reported outcome measures and digital psychometric scales, which capture perceived symptoms, self-efficacy, knowledge levels, and emotional well-being (22). To translate this multimodal data into actionable intelligence, machine learning models can be applied (22, 23). These models analyze integrated datasets to stratify patients by risk level and precisely identify individual gaps in knowledge, behavior, or clinical status, thereby establishing a targeted foundation for personalized intervention (23, 24).

### 3.2 Generating and delivering personalized interventions

Interventions are then tailored and delivered based on the assessment profile. A structured digital knowledge library enables content personalization: for instance, patients with poor medication adherence receive focused educational modules on pharmacotherapy, while those with reduced exercise capacity are guided through personalized breathing techniques and physical activity plans (25). Delivery is optimized through intelligent, context-aware systems that determine the timing (e.g., triggered by symptoms or daily routines) and channel (e.g., mobile app notifications, or virtual nurse interactions) of communication to enhance engagement (26). Furthermore, incorporating interactive elements—such as virtual peer-support communities and integrated telemedicine platforms for clinician consultation—strengthens the intervention by adding social and professional support, moving beyond purely automated education (27, 28).

### 3.3 Enabling a dynamic feedback mechanism

A dynamic feedback mechanism ensures the system remains responsive and adaptive. In the short term, real-time monitoring facilitates immediate guidance, such as alerts for missed medications or suggestions based on environmental triggers (29, 30). For long-term evaluation, the system analyzes trends in health outcomes—including symptom trajectories, exacerbation frequency, and behavioral changes—to assess intervention effectiveness and predict future risks (31, 32). Educational impact is further evaluated through periodic knowledge assessments and surveys of self-management behaviors (32). Critically, this feedback data is systematically reincorporated into the system. It refines the assessment algorithms, adjusts intervention content, and recalibrates delivery strategies, thereby completing a self-optimizing “assessment-intervention-feedback” loop that continuously improves the relevance and efficacy of COPD management (29, 33).

## 4 Challenges and countermeasures—key issues for implementation

### 4.1 Technical challenges

The effective implementation of this model faces significant technical hurdles, primarily concerning data integration and algorithmic reliability. Data heterogeneity presents a major barrier: information collected from diverse sources—such as portable devices, wearable sensors, environmental monitors, and patient self-reports—often differs in format, frequency, and quality (34). This necessitates standardized data processing and seamless system interoperability. Additionally, the reliability of algorithms, particularly those based on machine learning, requires careful consideration. Many models lack sufficient interpretability, making it difficult for healthcare professionals to trust their outputs (35). Their clinical validity should also be rigorously validated in real-world settings to avoid errors caused by data bias or overfitting (36).

### 4.2 User-level challenges

Successful adoption of the system depends on sustained patient engagement. Two key challenges emerge at this level. First, the “digital divide” may exclude older adults, a significant proportion of the COPD population, who may lack familiarity with or trust in digital tools (37). Second, maintaining long-term engagement is difficult; patients may lose motivation after initial enthusiasm, leading to discontinuation of use (38). Designing interventions that foster habitual use and sustained behavior change is therefore critical (39).

To promote sustained behavioral change rather than superficial engagement, digital COPD systems should be explicitly grounded in established psychological and behavioral science frameworks (40). The capability–opportunity–motivation–behavior model provides a structured lens for diagnosing barriers to effective self-management by evaluating whether patients possess the necessary skills, environmental support, and motivational drivers (40). Complementarily, self-determination theory underscores the importance of autonomy, competence, and social relatedness in fostering intrinsic motivation, which is more predictive of long-term adherence than extrinsic incentives (41). Operationalizing these theories within digital platforms may involve adaptive goal-setting algorithms, personalized feedback loops that reinforce incremental progress, and structured social reinforcement mechanisms such as peer support or clinician acknowledgment (42). Furthermore, just-in-time adaptive interventions enable context-sensitive prompts based on real-time symptom fluctuations or activity data, thereby delivering behavioral cues at moments of heightened receptivity (7). Insights from habit formation research further suggest that embedding consistent cue–routine–reward cycles into daily digital interactions can gradually transform deliberate self-management tasks into automatic behaviors, increasing the likelihood of sustained engagement over time (43).

By integrating behavioral theory with intelligent digital design, the system can move beyond passive information delivery toward an active, self-reinforcing engagement model (44, 45). Such an approach not only addresses motivational decline but also strengthens the psychological mechanisms underlying adherence, thereby enhancing the long-term viability and clinical impact of digital COPD management (30, 46).

### 4.3 Systemic challenges

From a broader healthcare system perspective, integration and regulation pose further obstacles. The closed-loop system should be compatible with existing clinical workflows and health information platforms, which often requires significant adjustments to infrastructure and professional roles (47). Healthcare providers may perceive these changes as disruptive or burdensome (48). Furthermore, strict adherence to data privacy and security regulations is essential, as health data are highly sensitive (49). Compliance with legal frameworks and ethical standards demands robust governance and protective measures.

Beyond regulatory compliance, governance structures should establish clear accountability pathways for algorithm-assisted clinical decision-making (50). For instance, when predictive models generate risk alerts, predefined escalation protocols should specify whether responsibility rests with automated systems, nurses, or attending physicians (50). Transparent documentation of algorithmic logic, validation datasets, and performance metrics is essential to foster clinician trust and reduce medicolegal ambiguity (51). Furthermore, ethical oversight committees may be required to conduct periodic audits of model fairness, ensuring that vulnerable subgroups are not disadvantaged by biased training data (52). Such proactive governance mechanisms transform abstract ethical principles into tangible, operational safeguards.

### 4.4 Potential countermeasures

Addressing these challenges requires a multi-layered and strategically coordinated approach spanning technical infrastructure, user engagement, clinical governance, and policy alignment.

At the technical level, interoperability should be strengthened through the adoption of standardized data exchange protocols and modular system architectures that enable seamless integration with existing electronic health records and clinical information systems (53). Such structural compatibility reduces fragmentation and enhances scalability across healthcare settings (53). Algorithm reliability should also be reinforced through rigorous external validation studies and prospective real-world pilot trials prior to large-scale deployment, thereby minimizing risks associated with data bias, overfitting, or context-specific performance degradation (54, 55).

Beyond technical robustness, implementation success depends heavily on user-centered strategies. A collaborative co-design process involving technologists, clinicians, and patients can improve usability, contextual relevance, and clinical credibility (56–58). At the user level, tiered onboarding programs—including structured digital literacy training for older adults and optional human coaching support—can reduce exclusion risks and improve confidence in system use (59). Rather than relying solely on automation, hybrid care models that combine digital monitoring with structured human oversight—such as periodic nurse-led reviews or case manager follow-ups—may enhance both patient safety and sustained engagement (60). By integrating automated alerts with professional interpretation, such models address concerns that fully autonomous systems may fail to detect nuanced clinical deterioration or subjective warning signs.

At the system and policy level, long-term sustainability requires supportive governance and reimbursement structures. Financial incentives and value-based reimbursement mechanisms can facilitate the incorporation of validated digital interventions into routine care pathways (61, 62). Clear regulatory standards for safety evaluation, data protection, and algorithm accountability are equally essential to promote institutional trust and ensure responsible innovation (62, 63). Together, these coordinated measures support not only technical feasibility but also ethical integrity and scalable implementation of digital COPD management systems.

## 5 Future perspectives: from a closed loop to a health ecosystem

The “assessment-intervention-feedback” closed loop represents a foundational model for digital COPD management. Its future evolution points toward a more integrated, person-centered, and proactive health ecosystem.

### 5.1 Technology integration trends

Advancements in technology will enable more sophisticated and responsive systems. Artificial intelligence, particularly natural language processing, will allow for conversational digital assistants capable of providing real-time, personalized patient education and support (64, 65). Furthermore, predictive analytics will evolve to anticipate subtle health deteriorations earlier, facilitating preemptive intervention (66). Immersive technologies such as virtual reality also hold promise for enhancing patient engagement in rehabilitation by creating simulated, interactive training environments that may improve both adherence and outcomes (67).

### 5.2 Directions for model expansion

The scope of digital management will broaden in two key directions. First, it will shift from managing COPD in isolation to addressing multiple chronic conditions concurrently (68). Future platforms should synthesize data across comorbidities—like cardiovascular or metabolic diseases—to deliver holistic and coordinated care plans. Second, the focus should expand from treatment to prevention (69). By identifying high-risk individuals through digital screening and wearables, these systems can establish early-intervention pathways, moving healthcare toward a more proactive, prevention-oriented paradigm (70).

### 5.3 Vision for value realization

The envisioned ecosystem aims to deliver significant value at both individual and systemic levels. For patients, it empowers sustained self-management, leading to improved quality of life, reduced exacerbations, and fewer hospital admissions (71, 72). For health systems, it optimizes resource allocation by automating routine monitoring, allowing clinicians to focus on complex care decisions (73). Ultimately, this evolution supports a fundamental shift from reactive, episodic treatment toward continuous, data-informed, and preventive health management (72).

## 6 Summary

The “assessment-intervention-feedback” digital closed-loop framework presented in this paper establishes a systematic approach for advancing COPD health education. Its primary contribution is facilitating a shift from standardized, episodic interventions to personalized, dynamic, and data-informed management. By enabling precise tailoring of educational content and allowing for continuous strategy refinement through real-world feedback, the framework enhances both the relevance and sustained effectiveness of patient support.

Successful implementation, however, requires meaningful collaboration across technical, clinical, and public health disciplines to ensure that technological tools are effectively integrated into care workflows. Future research should prioritize rigorous longitudinal studies to empirically evaluate the impact of this model on key clinical outcomes, such as exacerbation rates and quality of life, as well as on healthcare utilization and cost.

Equally important is addressing the digital divide to ensure equitable access. Proactive measures should be taken to include older and underserved populations, ensuring that the benefits of digital innovation are broadly shared. Ultimately, this framework supports the evolution of COPD care toward a more proactive, preventive, and patient-centered health ecosystem.

## Data Availability

The original contributions presented in the study are included in the article/supplementary material, further inquiries can be directed to the corresponding author.
